# Prison and forensic mental health staff after suicides in their care. A narrative review of international and German national evidence

**DOI:** 10.3389/fpsyt.2024.1400604

**Published:** 2024-06-13

**Authors:** María Isabel Fontao, Jan Bulla, Thomas Ross

**Affiliations:** ^1^ Department of Psychology, University of Konstanz, Konstanz, Germany; ^2^ Forensic Psychiatry and Psychotherapy, Reichenau Psychiatric Center, Reichenau, Germany; ^3^ Department of Forensic Psychiatry and Psychotherapy, Ulm University, Ulm, Germany

**Keywords:** prisoner suicide, patient suicide, impact, prison staff, mental health staff, forensic psychiatry

## Abstract

There is a growing body of international research investigating the impact of patient suicide on mental health professionals. The experience of losing a patient to suicide can have a significant and, in some cases, long-lasting (negative) impact on mental health professionals. However, the nature and extent of the impact on prison staff or forensic mental health professionals in particular is less clear. This narrative review summarises both quantitative and qualitative studies and key findings in this area, focusing on the above professions. A literature search was conducted using PsychInfo and Google Scholar, covering the period from 2000 onwards. The vast majority of findings relate to mental health professionals in general. We were unable to identify any published reports on the responses of forensic psychiatric staff. The majority of identified studies in the prison context are qualitative. Studies from German-speaking countries are particularly scarce in both the prison and mental health contexts. We conclude that there is a profound lack of knowledge about the impact of client/patient suicide on the subgroups of (German) prison and forensic psychiatric staff. Clearly, more research is needed on both the nature and extent of the impact, as well as on the specific organisational and supportive factors that help to mitigate the negative effects of suicide.

## 1 Introduction

Compared with the general population, mental health and prison staff are at increased risk of being exposed to attempted or completed suicide in their professional environment (1, 2). Between 31.5% and 92% of mental health professionals have been exposed to patient or client suicide at least once in their careers (3). In a representative sample of sworn officers working for the Massachusetts Department of Correction (*N* = 317), participants had experienced an average of 1.04 (*SD* = 2.46) inmate suicides; 35% had experienced two or more (4). Other international and German national data on suicide suggest that suicide in prisons is common (e.g. 5–7).

Exposure to suicide in a professional setting can have far-reaching and lasting consequences, some of which affect professional and personal well-being, risk of burnout and mental health (8). In recognition of the problem, guidelines and recommendations have been developed either for internal use within organisations or for wider professional audiences (for recommendations in England, see Royal College of Psychiatrists report CR 234, 9). These and other guidelines aim to reduce distress following patient suicide and prevent long-term effects at the personal level, and to ensure effective risk management and promote staff retention by protecting against burnout at the organisational level (10). In Germany, the manuals and catalogues of measures that are usually available in professional institutions for staff following a patient or prisoner suicide are typically organisational or workflow oriented, but widely accepted in general mental health practice (e.g., 11). However, the general guidelines and recommendations for managing staff after a patient or client suicide do not seem to take into account the particular risk that forensic staff face in their work environment.

To summarise the available evidence, a narrative literature review was conducted with the following key questions:

1. To what extent is there scientific evidence on the impact of prisoner or patient suicide on the work and/or personal well-being of prison and forensic psychiatric staff?
2. Is there specific published evidence for forensic mental health professionals and/or prison staff in Germany?

## 2 Methods

Literature searches were conducted using the PsycInfo database and Google Scholar. The search strategy was based on the review by Sandford et al. (10); the search terms for PsycInfo are shown in **Table 1**. Only literature in English or German was considered. The search yielded 3408 hits, of which the first 250 hits were screened using title and abstract. The results in PsycInfo were supplemented by text-based searches in Google Scholar, all without filters. Here, too, the first 250 hits were checked by title and summary.

**Table 1:** Search terms for the literature review.

1. Prisoner/Patient suicide	2. Profession	3. Impact
prisoner* suicide* OR inmate* suicide*	correctional officer* OR prison employee* OR prison guard* OR corrections officer*	reaction* OR impact* OR effect*
patient suicide OR client suicide OR patient death by suicide	professional* OR practitioner* OR clinician* OR nurse* OR worker* OR therapist* OR psychologist* OR psychiatrist* OR counsellor* OR GP	reaction* OR impact* OR effect*

Searches (columns 1–3) were combined using the operator “AND”.

(*): truncation wildcard to find multiple endings.

The selection criteria were as follows: Original empirical research and review papers from 2000 onwards were considered for inclusion. Only peer-reviewed original empirical studies that explicitly examined the impact of suicide were included. Studies whose specific main topic was not the impact of suicide, but related topics (e.g., impact of self-harm other than suicide or non-suicide-related emotional distress among mental health/prison staff; description or evaluation of suicide prevention or postvention) were not included.

## 3 Results

In accordance with the selection criteria, six original research papers from the prison context and three original research papers from Germany (general mental health) were included in this mini review. We identified ten reviews on the impact of patient suicide among (mental health) professionals, but were unable to find studies that investigated the impact of patient suicide in forensic psychiatric context. The characteristics of the included original studies (country, research design and methods, sample and setting) and the identified review papers (number of papers included and research design, sample size) are presented in the **Supplementary Materials**.

### 3.1 Prison

In recent decades, the negative effects of imprisonment on the mental health of prisoners have been empirically investigated, but less attention has been paid to the psychological effects of working in a secure environment on prison staff (4). A recent review of the mental health of prison officers, based on six published studies (with sample sizes ranging from 65 to 3,274) and one unpublished research report (*N* = 3,599), estimated the prevalence of PTSD at 15 – 34%, anxiety at 24 – 25% and depression at 24 – 60% (12). Six original research articles that specifically addressed the impact of prisoner suicide on the mental wellbeing and mental health of prison staff were considered. Wright et al. (13) investigated the incidence of PTSD among prison officers (*N* = 49) who had dealt with a prisoner’s self-inflicted death in custody 3–7 months prior to the study, and found that 36.7% of the 49 study participants scored above the cut-off point on the Trauma Factor of the Traumatic Symptom Inventory. For the PTSD group, the only predictor of traumatic symptoms was previous exposure to self-inflicted death.

Five out of six articles included in this section used qualitative methods. In order to accurately reflect the unique characteristics of these findings, we present the studies individually. Barry (14, 15 conducted 17 semi-structured interviews to explore how prison officers deal with emotional experience following the death of a prisoner. Using thematic analysis, the author describes and maps the characteristics of the emotional processing during the call (emotional neutrality, detachment), and behaviour immediately after the incident (humour to regulate emotional display, controlled expression of empathy) and after the incident (mitigating emotional impact, seeking support at work, separating home and work). An important implication of dealing with the death of an inmate was that many prison staff members may feel obliged to act and feel according to their perceived role within the prison system and the rules set by institutional and organisational management.

Using semi-structured interviews with 11 prison officers, Lavrič et al. (16) explored the following topics: prior expectations of encountering inmate suicidal behaviour, the event of the inmate suicide, feelings/thoughts/psychological distress upon encountering suicide, coping mechanisms and support in coping, relationships with colleagues, understanding of their role in preventing inmate suicidal behaviour, and attitudes towards suicide. Using a grounded theory approach, the authors developed a model describing different aspects of correctional officers’ experiences of inmate suicidal behaviour based on correctional officers’ beliefs about inmate suicidal behaviour, officers’ preparedness to deal with inmate suicides, and officers’ understanding of their role in interventions to prevent inmate suicidal behaviour.

Sweeney et al. (17) interviewed nine prison officers about their thoughts, feelings, coping strategies and support following an incident of suicidal behaviour. Thematic analysis was used to create a thematic map of key categories of correctional officer culture: limited support, feeling unqualified, under-resourced, minimising negative emotions, and positivity. The first incident of suicidal behaviour was described as the most distressing. Emotional consequences included fear, anxiety, nightmares and flashbacks. Furthermore, the authors observed that the reluctance to express emotions is a common phenomenon in the prison context. Participants perceived avoidance coping as a necessity due to the limited support available and cultural barriers.

Ricciardelli et al. (18) examined the impact of exposure to attempted and completed suicide among 43 correctional officers, focusing on their emotional responses and available support. The participants had often experienced at least one suicide-related incident. Using a semi-grounded constructed approach, the interview transcripts were coded for emergent themes in a multi-step process. A key finding was that the participants tended to feel underprepared or unprepared to support inmates at risk of suicidal behaviour. According to the authors, the working culture values toughness, which may inhibit talking about emotions and mental health after critical incidents. There appears to be a divide among prison officers about the acceptability of seeking support after a critical incident, with some officers preferring to cope alone and others choosing to discuss their feelings with colleagues. The majority of participants felt that their management did not care about their mental wellbeing.

### 3.2 Forensic psychiatry and mental health

#### 3.2.1 Forensic psychiatry

No original studies were identified that specifically addressed the impact of patient suicide on staff in forensic hospitals.

#### 3.2.2 Mental health (reviews)

We identified ten narrative reviews of the impact of patient suicide on mental health professionals and related professional groups. Recent studies, in particular, are based on systematic searches of specialist databases and are therefore considered to be an accurate reflection of the current state of research. These studies examined the impact of patient suicide on specific professional groups (nurses: 19, 20; psychiatrists: 21, 22) or across professional groups (nurses, psychiatrists, psychologists, GPs, social workers, firefighters, police and prison officers; 3, 10, 23–26). The majority of these reviews included studies that used quantitative (questionnaires and scales, statistical analysis) and qualitative (interviews, content analysis) methods.

Overall, more recent reviews (publication year up to 2021) confirm the findings of earlier reviews. Key findings on the impact of patient suicide on mental health workers are presented below, based on the recent comprehensive reviews by Lyra et al. (3), MacGarry et al. (26) and Sandford et al. (10). Other criteria for the decision to report these three reviews were the specificity of the target population (mental health professionals, including different professions) and the theoretically sound categories used in the review (e.g., emotional and professional impact, factors increasing vulnerability, risk assessment and management).

Among the reported original studies that mainly focused on general mental health samples, the reviews by MacGarry et al. (26) and Sandford et al. (10) also included five and three original studies, respectively, that investigated mixed samples from mental health, care homes and, remarkably, forensic psychiatry and prisons (27–31). However, there were no original studies that investigated only forensic psychiatric mental health professionals.

The impact of patient suicide can be categorised as ‘personal’ and ‘professional’ (life, wellbeing or practice). *Personal reactions* can show as guilt, blame, shock, anger, sadness, anxiety and grief (3, 10), rumination, self-monitoring, sleep disturbance, exhaustion and loss of appetite (10), feelings of failure, powerlessness and responsibility for the suicidal behaviour (26). Emotional distress following exposure to suicide can be prolonged and lead to burnout; trauma reactions including intrusive images, panic attacks and nightmares have also been reported (3, 10, 24, 26). Other responses were acceptance and understanding of the patient’s decision and an increased awareness of their professional ability to prevent suicide (10). However, the experience of patient suicide can also trigger suicidal ideation in clinicians (10).

*Professional responses* involve increased awareness of suicide risk and a defensive approach to suicide risk (e.g. more conservative/detailed record keeping, greater attention to legal issues), reduced professional confidence in the own judgement and decision making and increased consultation with colleagues (10), fear of publicity and litigation, increased referrals to psychiatrists, and grief at work (3). Professional responses can be described at individual and organisational levels and be assessed as positive (e.g. improved competence in dealing with suicidal behaviour, collaborative risk management) or negative (e.g. negative changes in clinical practice, feeling controlled by an increasingly unpleasant working environment) (26).

Findings on the proportion of respondents reporting minimal, moderate or severe effects of patient suicide were inconsistent. Sandford et al. (10) included ten studies that reported on the duration of the initial impact following patient suicide. There was considerable variation in how the duration was recorded and reported, which may partly explain why the measured duration of effects following exposure to suicide ranged from one week to six months or longer. All studies agreed that the (negative) effects tended to diminish over time (10). The time elapsed between the suicide and the assessment of its effects varied widely not only within, but also between studies (10).

The intensity of the adverse effects of suicide exposure is associated with personal, organisational, relational (staff-patient interaction) and role-related factors. Higher levels of education and training have been described as protective, and being female and younger than 50 may increase the impact of patient suicide (26). Clinician perception of responsibility for the death, unpredictability, method of suicide and proximity were associated with increased impact (10).

#### 3.2.3 Mental health (Germany)

In Germany, Wurst et al. (32–34 investigated therapists’ reactions to a patient’s suicide. Wurst et al. (32) investigated how *N* = 172 therapists from southern Germany and Switzerland (*n* = 125 in private practice; *n* = 47 in institutional settings; the professionals in institutional settings included 26 psychiatrists in training, 7 senior psychiatrists, and 14 psychologists) reacted to a patient’s suicide over time and which factors contributed to the reaction. Common immediate reactions among all therapists were sadness, guilt, shame, shock, feelings of inadequacy and disbelief, with all emotions fading over a period of six months after the incident. Compared to psychiatrists and psychologists in training, experienced therapists tended to be less affected and cope better overall, but no difference in overall distress was observed between professional groups. Wurst et al. (33) again published the results of a survey of all psychiatric clinics in general hospitals in Germany (185 at the time). The total sample (*N* = 179 from 77 clinics) included 69 psychiatrists in training, 62 senior psychiatrists, 42 psychologists and 5 members of other professions (1 missing). The therapists’ emotional response to a patient’s suicide was measured after two weeks and after six months. Three out of ten therapists experienced severe distress as a result of a patient’s suicide, and the intensity of distress predicted therapists’ emotional reactions and behavioural change. The authors concluded that more specific and intensive help should be provided to these professionals. The above findings (32, 33) were tested for robustness and confirmed two years later (34). The sample comprised *N* = 226 therapists from 93 hospitals including 95 psychiatrists in training, 69 senior psychiatrists, 56 psychologists and 5 members of other professions (1 missing). Approximately 40% of therapists experienced severe distress following a patient suicide. Overall distress was used to identify subgroups that might need individualised postvention. Highly distressed therapists were more likely to be female, to feel less supported by their colleagues and institution, to have a high fear of litigation, to be more afraid of the reaction of the deceased’s relatives, to be more cautious, and to be unable to continue working as usual. The authors suggested that this subgroup of therapists needs individual approaches/therapy in addition to general approaches such as suicide conferences and supervision.

## 4 Discussion and future directions

### 4.1 Impact of suicide on mental health professionals and prison staff

The literature clearly suggests a significant short-term (and in some cases long-term) negative impact of patient suicides on mental health professionals. The existing evidence on the impact of suicide on first responders, e.g. nurses, social workers, firefighters and police officers, is significantly less than for psychologists and psychiatrists, which calls for further research into the impact of exposure on suicide among members of these professions (3). Based on our own findings, forensic psychiatric and prison staff can be added to this list. Compared to the published findings on the impact of patient suicide on mental health professionals, research on the impact of prisoner suicide on the mental health of prison staff is scarce. Only in recent years has empirical evidence accumulated on the negative effects of the prison environment on prison staff, showing that exposure to violence in the workplace increases the risk of mental health symptoms. However, the specific consequences of prisoner suicide and its predictors or moderators have rarely been investigated. Five papers (27–31) referred to by MacGarry et al. (26) and Sandford et al. (10) examined mixed samples that included professionals working in prison and forensic psychiatry. However, the results for these (small) subsamples were not reported separately.

Focusing on Germany, there is a noticeable lack of empirical evidence on the impact of prisoner or patient suicide on both prison and forensic mental health professionals. The available evidence focuses on German mental health professionals in general, was conducted in the 2010s, and does not appear to have been continued or extended to forensic psychiatric samples (32–34).

### 4.2 Prevention and postvention

The evidence shows that more should be done to prepare and support mental health professionals in the event that they lose a patient or inmate to suicide. Further research into the moderating factors of the most common personal reactions (blame, guilt and feelings of failure, etc., see section 3.2.2) may help to develop specific interventions to protect staff members against the impact of patient suicide in professional settings. According to Sandford et al. (10), the emotional impact of patient suicide on professionals may have a similar impact as other traumatic events. Prevention guidelines suggest that those with subclinical symptoms should be monitored in the short term and those with clinical symptoms should be offered trauma-focused therapy (for detailed recommendations, see the Royal College of Psychiatrists report CR 234, 9). There are several other types of support that can help minimise negative effects (e.g., 10, p. 289): informal support from colleagues, peers, family or friends has been described as most helpful. Formal support often includes supervision (by the organisation), but the evidence for its effectiveness is more mixed. Team meetings may be helpful for some, but not for others. Case reviews may also be helpful in some, but not all cases (not helpful in cases where the organisation’s actions are perceived as insensitive or persecutory) (10). Relationship factors (e.g. duration and/or quality of relationship with patients who have committed suicide, or quality of relationship) may either increase or decrease negative effects (26).

## 5 Limitations and strengths

The aim of this mini review was to gain an initial understanding of the impact of prisoner or patient suicide on staff in forensic institutions. A major strength is the comprehensive nature of this review, which outlines the research evidence on the experiences of prison and mental health staff working with people at risk of suicide in custodial settings and in the mental health sector. It also highlights the personal and professional impact of these events, the factors that might affect the impact, and how professionals could be supported.

The limitations of this review relate to the search strategy and the selection criteria. Because only published articles in English and German in PsycInfo and Google Scholar were considered for inclusion, potentially relevant research work that is not indexed in these databases could not be found. The data collection and analysis methods of the original studies are heterogeneous, and we did not use a structured scale to assess their methodological quality. The legal requirements for placement of offenders in prisons and in secure hospitals and the organizational structures in these high security environments can vary considerably from country to country. This may limit the generalizability of the results reported here.

For the prison context, future research should re-examine the findings of qualitative research using standardised methods and larger samples. There is a substantial body of scientific evidence on the personal, professional and/or organisational impact of suicide on mental health staff in general, but no specific findings on forensic mental health staff. Future studies should fill this research gap. A systematic review using a wider range of search terms and databases could help expand the results reported in this mini review. Our findings will be of particular interest to prison and forensic mental health services and policy makers who are responsible for designing, implementing and funding projects in the areas addressed here.

## Author contributions

MF: Conceptualization, Investigation, Methodology, Supervision, Validation, Writing – original draft, Writing – review & editing. JB: Validation, Writing – review & editing. TR: Conceptualization, Investigation, Methodology, Validation, Writing – original draft, Writing – review & editing.
