# Supplementary material for: Prison and forensic mental health staff after suicides in their care. A narrative review of international and German national evidence
Source: Front Psychiatry. 2024 Jun 13;15:1400604. doi: 10.3389/fpsyt.2024.1400604 (PMC11209187; doi:10.3389/fpsyt.2024.1400604)
Supplement: Supplementary file 1 [file Table_1.docx]

Supplementary Material

# Supplementary Tables

1.1 Impact on prison / correctional officers of inmate suicide

| **Study, country** | **Research design, methods** | **Sample, number, setting** |
| --- | --- | --- |
| Barry (2017, 2020)  Ireland | Qualitative  Semi-structured interview | Prison officers  *N* = 14 (2017), *N* = 17 (2020)  Prison |
| Lavrič et al. (2022)  Slovenia | Qualitative  Semi-structured interview | Correctional officers  *N* = 11  Prison |
| Ricciardelli et al. (2020)  Canada | Qualitative  Semi-structured interview | Correctional officers  *N* = 43  Prison |
| Sweeney et al. (2018)  United Kingdom | Qualitative  Semi-structured interview | Prison officers  *N* = 9  Prison |
| Wright et al. (2006)  United Kingdom | Quantitative  Standardized questionnaires: Trauma Symptom Inventory (TSI), Locus of Control of Behaviour Scale (LCBS), Problem-Solving Style Questionnaire (PSQ), Life Orientation Test (LOT), Significant Others Scale (SOS) | Prison officers  *N* = 49  Prison |

1.2 Impact on mental health professionals of patient suicide: Reviews

| **Review** | **Number of papers included** | **Research design of original studies** | **Sample, range of sample sizes** |
| --- | --- | --- | --- |
| Causer et al., 2019 | 12 articles reporting on 11 studies | Qualitative | Psychiatric nurses, school counsellors, clinical psychologists, general practitioners, school teachers, nurses in general hospital, mental health social workers  *N* = 5 – 198 |
| Fothergill et al., 2004 | 23 | Quantitative | Focus on psychiatrists. Other mental health professionals (clinical psychologists; psychiatric / mental health nurses; social workers; occupational therapists; mental health professionals)  Sample sizes not reported |
| Leaune et al., 2019 | 22 | Quantitative, qualitative | Psychiatric trainees, child and adolescent psychiatric trainees  *N* = 9 – 203 |
| Lyra et al., 2021 | 25 | Quantitative, qualitative | Mental health (mental health professionals, psychiatrists, psychologists, nurses, counsellors, social workers), first responders (police officers, firefighters)  *N* = 6 – 1027 |
| MacGarry et al., 2022 | 20 | Quantitative, qualitative, mixed-methods | Mental health (nurses, psychiatrists, psychologists, social workers, general practitioners, etc.)  *N* = 6 – 713 |
| Malik et al., 2022 | 10 | Qualitative | General practitioners, psychiatrists, (mental health) nurses  *N* = 2 – 198 |
| Sandford et al., 2021 | 54 | Quantitative, qualitative, mixed-methods | Mental health professionals, psychiatrists, psychologists, psychotherapists, social workers, general practitioners, counsellors, nurses  *N* = 8 – 666 |
| Séguin et al., 2014 | 37 | Quantitative, qualitative | Psychologists, psychiatrists, psychiatric nurses, social workers, general practitioners  *N* = 1 – 531 |
| Talseth & Gilje, 2011 | 26 | Quantitative, qualitative, mixed-methods | Nurses (psychiatric, oncology, emergency, etc.)  *N* = 4 – 454 |
| Valente & Saunders, 2022 | 8 | Quantitative, qualitative | Focus on nurses. Other mental health professionals (psychiatry residents / multidisciplinary team, psychiatrists, clinical psychologists, psychology interns)  *N* = 1 – 131 |

1.3 Impact on mental health professionals of patient suicide: Studies in Germany

| **Study, country** | **Research design, methods** | **Sample, number, setting** |
| --- | --- | --- |
| Wurst et al., 2010  Germany | Quantitative  63-item questionnaire: Therapist and patient characteristics, treatment, risk assessment, support | Therapists (psychiatrists, psychologists)  *N* = 172  Private practice (*n* = 47), Institutional setting (*n* = 125) |
| Wurst et al., 2011  Germany | Quantitative  63-item questionnaire: therapist and patient characteristics, treatment, risk assessment, support | Therapists (psychiatrists, psychologists, other)  *N* = 179 (from 77 clinics)  Institutional setting |
| Wurst et al., 2013  Germany | Quantitative  63-item questionnaire: therapist and patient characteristics, treatment, risk assessment, support | Therapists (psychiatrists, psychologists, other)  226 (from 93 hospitals)  Institutional setting |
